# Terlipressin for the Prevention and Treatment of Renal Decline in Hepatorenal Syndrome: A Drug Profile

**DOI:** 10.3390/gastroent14040031

**Published:** 2023-09-28

**Authors:** Ahlam Ayyad, Rami A. Al-Horani

**Affiliations:** 1 Division of Clinical and Administrative Sciences, College of Pharmacy, Xavier University of Louisiana, New Orleans, LA 70125, USA; 2 Division of Basic Pharmaceutical Sciences, College of Pharmacy, Xavier University of Louisiana, New Orleans, LA 70125, USA

**Keywords:** hepatorenal syndrome, acute kidney injury, terlipressin, vasopressin analogue

## Abstract

Hepatorenal syndrome stands as one of several potential triggers of acute kidney injury in individuals grappling with either acute or persistent liver ailments. The nature of the decline in kidney function has led to the identification of two variants of hepatorenal syndrome. In cases where terlipressin therapy is accessible, the initial approach involves administering terlipressin alongside albumin. Terlipressin, a synthetic analog of vasopressin, boasts double the preference for vasopressin V1 receptors compared to V2 receptors. The FDA granted approval to terlipressin in September 2022, demonstrating its intrinsic activity, although a significant portion of its function arises from its transformation into lysine vasopressin. This article provides a comprehensive examination of terlipressin’s various pharmacodynamic and pharmacokinetic facets, as well as its clinical utility, aiming to keep the scientific community well informed about its safe and efficient utilization.

## 1. Introduction

Hepatorenal syndrome emerges as a plausible contributor to acute kidney injury among patients grappling with either acute or chronic liver diseases. Those affected typically exhibit portal hypertension due to conditions such as cirrhosis, severe alcoholic hepatitis, or metastatic tumors. This syndrome, however, can also manifest in individuals experiencing fulminant hepatic failure [1-4]. The condition represents the final stage of a series of reductions in kidney perfusion induced by increasingly severe hepatic injury. Diagnosing hepatorenal syndrome involves systematically excluding alternative potential causes and is associated with an unfavorable prognosis. The shifts in hemodynamics and the decline in kidney function seen in cirrhosis primarily stem from arterial vasodilation within the splanchnic circulation, a consequence of portal hypertension. This vasodilation is thought to be induced by an increased production or activity of vasodilatory agents, predominantly within the splanchnic circulation, where nitric oxide is believed to play a crucial role [1-4]. A prospective study evaluating nonazotemic patients with ascites and cirrhosis provided insights into the frequency of hepatorenal syndrome occurrence [5]. Within one and five years, hepatorenal syndrome developed in 18% and 39% of cases, respectively. Moreover, hepatorenal syndrome is a prevalent occurrence among patients with acute liver disease. For instance, in a study involving patients with alcoholic hepatitis, approximately 28% of individuals experienced hepatorenal syndrome [6].

In patients who have established or clinically evident acute or chronic liver disease, the emergence of hepatorenal syndrome is characterized by a gradual increase in levels of serum creatinine. The urine sediment typically appears normal or shows minimal proteinuria (less than 500 mg per day), and the rate of sodium excretion is very low. The patient may experience nonoliguria or oliguria, depending on the severity and duration of the condition [1-3,5,7]. It is noteworthy that a substantial number of individuals with hepatorenal syndrome exhibit nonoliguric tendencies, and several studies have documented daily urine volumes exceeding 400 mL. A marked decrease in urine output is frequently observed only a few days prior to the individual’s death [8,9]. Additionally, the increase in serum creatinine levels can be as minimal as 0.1 mg/dL per day, occasionally punctuated by phases of stabilization or slight amelioration. Furthermore, the urine sediment may display several irregularities, including hematuria stemming from instrumentation of the bladder and underlying coagulopathy, as well as granular casts arising due to hyperbilirubinemia.

Categorizing hepatorenal syndrome based on the rate of kidney function decline has led to the recognition of two distinct forms [1,7,10,11]. One of these forms, a more severe manifestation, is referred to as hepatorenal syndrome–acute kidney injury (HRS-AKI) or traditionally termed type 1 hepatorenal syndrome. It is characterized by an absolute increase in serum creatinine of ≥0.3 mg/dL within 48 h and/or urine output ≤ 0.5 mL/kg body weight for ≥6 h or a percent increase in serum creatinine of ≥50% using the last available value of outpatient serum creatinine within 3 months as the baseline value. The other form is recognized as hepatorenal syndrome–non-acute kidney injury (HRS-NAKI) or traditionally labeled type 2 hepatorenal syndrome. In this variation, the decline in kidney function is less severe compared to HRS-AKI. The defining clinical feature of the second hepatorenal syndrome type is the presence of ascites that remains unresponsive to diuretic treatment. Other remarkable criteria in HRS-NAKI include the estimated glomerular filtration rate (eGFR) is to be less than 60 mL/min per 1.73 m^2^ for <3 months (in acute kidney disease) or for ≥3 months (in chronic kidney disease) in the absence of other (structural) causes, and the percent increase in serum creatinine is to be <50% using the last available value of outpatient measurement within three months as the baseline value [12]. Overall, the diagnosis of hepatorenal syndrome is made by excluding other potential causes of acute or subacute kidney injury. Only after ruling out these other causes can the possibility of hepatorenal syndrome be considered.

## 2. Treatment of Hepatorenal Syndrome

A variety of strategies are exploited to treat/manage hepatorenal syndrome. These methods encompass endeavors such as recovering from alcoholic hepatitis, employing effective antiviral therapy for decompensated hepatitis B, achieving recovery from acute hepatic failure, and considering the possibility of liver transplantation [13-15]. Yet, in instances where immediate improvement in liver function is not attainable, addressing the acute kidney injury linked to hepatorenal syndrome necessitates medical intervention. The discontinuation of antihypertensive agents, including beta blockers, is recommended for all individuals with hepatorenal syndrome. For patients within intensive care units, initial treatment is proposed to encompass a combination of norepinephrine and albumin. Regarding individuals with hepatorenal syndrome situated outside the intensive care setting, the availability of specific pharmaceuticals assumes a pivotal role in dictating the treatment course. In scenarios where terlipressin therapy is accessible, the initial strategy involves combining terlipressin with albumin, as has been advised. In cases where terlipressin is not an option, an alternative approach involves a combination of midodrine, octreotide, and albumin. In select cases where medical interventions prove inadequate despite the mentioned regimens and when suitability criteria are met, the transjugular intrahepatic portosystemic shunt has demonstrated some favorable outcomes. For patients who fail to respond to the mentioned therapies, experience severely impaired kidney function, and either qualify for liver transplantation or have a reversible liver injury with a good chance of survival, the consideration of dialysis arises. Dialysis can serve as a bridge toward liver transplantation or the potential recovery of liver function [16].

## 3. Vasopressin: Background

**Figure 1.**
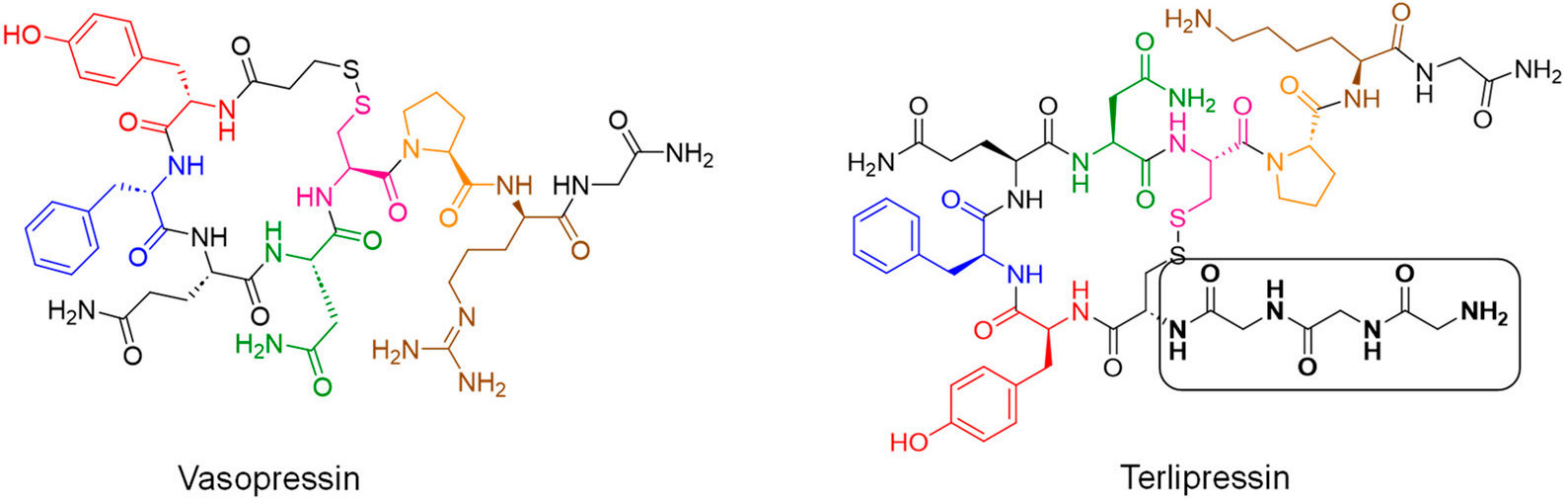
Chemical structures of vasopressin and terlipressin. Similar amino acids are color-coded.

Vasopressin (Figure 1), also known as antidiuretic hormone (ADH) or arginine vasopressin (AVP), is a 9-amino-acid oligopeptide that is synthesized in the hypothalamus and stored in the posterior pituitary gland. Its crucial functions include regulating osmotic balance, sodium homeostasis, blood pressure, and kidney function. In the kidney, vasopressin stimulates the expression of water transport proteins in the late distal convoluted tubule and collecting duct, leading to increased water reabsorption. Alterations in vasopressin secretion or tissue sensitivity can result in various diseases. At the molecular level, vasopressin exerts its physiological effects by interacting with three subtypes of vasopressin receptors (V receptors). The V1 receptors are found on vascular smooth muscle and other cells, including hepatocytes. The V2 receptors are predominantly expressed on the basolateral membrane of the distal convoluted tubule and collecting ducts in the kidney. The V3 receptors mediate the effects of vasopressin on the central nervous system [17-19].

On one hand, vasopressin binding to V1 receptors activates G protein, which subsequently actives phospholipase C (PLC), leading to the production of inositol triphosphate (IP-3) and diacylglycerol (DAG) as second messengers. IP-3 increases the release of intracellular calcium from the endoplasmic reticulum. DAG and calcium activate protein kinase C, which triggers a signaling phosphorylation cascade, resulting in vasoconstriction, increased peripheral resistance, and increased blood pressure [20-22]. On the other hand, vasopressin binding to V2 receptors leads to the activation of adenylate cyclase, which subsequently increases the production of the second messenger cyclic AMP (cAMP). The latter activates protein kinase A, an intracellular phosphorylating enzyme, which ultimately causes intracellular aquaporin-2 (AQP2) to be phosphorylated and to translocate to the apical membrane. AQP2 is a water channel that passively allows renal water reabsorption, as determined by the osmotic gradient established by urea and sodium chloride. Thus, vasopressin’s action enables our body to conserve water in conditions of dehydration or substantial loss of blood [17-19]. Importantly, vasopressin is a stress hormone that is released in response to stress stimuli, such as surgery, shock, pain, and syncope. Shock conditions such as hypovolemia cause an increase in the release of vasopressin to maintain organ perfusion [17-19]. There are several analogs of vasopressin such as lypressin, argipressin, desmopressin, felypressin, phenypressin, ornipressin, selepressin, but this article focuses on terlipressin.

## 4. Terlipressin: Chemistry

Terlipressin (Figure 1), a prodrug and synthetic analogue of natural vasopressin, is a 12-amino-acid oligopeptide. It consists of a cyclic hexapeptide domain, including Tyr, Phe, Gln, and Asn, along with two Cys residues linked by a disulfide bond. The N-terminus has a tripeptide sequence of three Gly residues, while the C-terminus contains a tripeptide of Gly, Lys, and Pro. This configuration gives rise to the chemical name N-[N-(N-glycylglycyl)-glycyl]-8-L-lysine-vasopressin, also known as the triglycyllysine derivative of vasopressin [23]. When administered intravenously, terlipressin, supplied as a sterile, lyophilized, acetate salt, is biotransformed into its active metabolite, lysine vasopressin (LVP). This transformation occurs through the cleavage of the three Gly residues from the N-terminus. The molecular formula of the free base of terlipressin is C_52_H_74_N_16_O_15_S_2_, and its average molecular weight is 1227.38 [23].

## 5. Terlipressin: Mechanism of Action

Terlipressin possesses vasoconstrictive, antihemorrhagic, and antidiuretic properties. Terlipressin appears to act both as a pharmacologically active drug as well as a prodrug for LVP. Upon IV administration, terlipressin can be biotransformed into LVP, which has variable binding affinities to vasopressin receptors (V1–V3). As a better agonist for V1 receptors, terlipressin increases systemic vascular resistance and decreases portal pressure. Therefore, terlipressin is believed to enhance renal blood flow in individuals afflicted with hepatorenal syndrome through a dual mechanism by reducing portal hypertension and blood circulation within portal vessels, while simultaneously augmenting effective arterial volume and mean arterial pressure [24-26]. Binding to V3 receptors induces adrenocorticotropic hormone secretion. Importantly, terlipressin has a lesser effect on V2 receptors, binding to which promotes renal water reabsorption [24-28].

## 6. Terlipressin: Pharmacokinetics

Given the protein nature of the drug, the drug is used parenterally via intravenous injection. Terlipressin and its major metabolite, lysine vasopressin, exhibit linear pharmacokinetics in healthy subjects. Their plasma concentrations increase proportionally with the administered doses. The volume of distribution of terlipressin is 6.3 L and 1370 L for its metabolite (lysine vasopressin). Terlipressin clearance is 27.4 L/hr, and its metabolite’s clearance is 318 L/hr. No dose-dependent changes in the elimination rate constant of terlipressin were observed in healthy subjects. Nevertheless, the clearance of terlipressin, but not lysine vasopressin, increases with body weight in the intended patients. The terminal half-life of terlipressin is 54 min, whereas the terminal half-life of lysine vasopressin is 3.0 h [22,29,30].

Terlipressin is metabolized by the cleavage of the three glycine residues of the N-terminus by tissue peptidases, leading to the release of its pharmacologically active metabolite lysine vasopressin. Lysine vasopressin is cleaved by different tissue peptidases. Terlipressin is not metabolized in blood or plasma. The metabolism of terlipressin is not affected by the disease state or the concurrent administration of other drugs. Owing to their extensive metabolism, less than 1% of terlipressin and less than 0.1% of lysine vasopressin is excreted in urine in healthy subjects. Age, gender, creatinine clearance, cirrhosis, serum alkaline phosphatase, serum aspartate aminotransferase, serum alanine aminotransferase, and total bilirubin do not appear to have a clinically significant effect on the clearance of terlipressin or lysine vasopressin. In vitro studies in human liver microsomes indicated that terlipressin is not a time-, direct-, or metabolism-dependent inducer or inhibitor of the hepatic cytochrome P450 enzymes. Furthermore, terlipressin is not an inhibitor or a substrate of the human ATP-binding cassette or solute carrier transporters. Therefore, no significant drug–drug interactions are expected with terlipressin [22,29,30].

## 7. Terlipressin: Clinical Trials

Several investigations have been conducted on patients with cirrhosis and hepatorenal syndrome associated with acute kidney injury (HRS-AKI) to assess the effectiveness of terlipressin therapy (as indicated in Table 1). The response to terlipressin in achieving hepatorenal syndrome reversal, defined as a decrease in serum creatinine to ≤1.5 mg/dL (≤133 μmol/L), has been observed to vary between 35% and 80%. Both HRS-AKI and hepatorenal syndrome not associated with AKI (HRS-NAKI) seem to exhibit similar response rates to terlipressin. However, HRS-NAKI patients treated with terlipressin have a higher risk of recurrence (approximately 50%) compared to HRS-AKI patients (8%). When terlipressin is used in combination with albumin, it has been found to be highly effective in reversing HRS-AKI, with response rates as high as 80%. Furthermore, there is clear evidence of improved survival when terlipressin and albumin are administered together, compared to a placebo (or no treatment), albumin alone, or terlipressin alone. However, no survival benefit has been reported when compared to other vasoconstrictors. In comparison to other treatments like midodrine/octreotide/albumin, the combination of terlipressin and albumin has demonstrated superior effectiveness in improving renal function in HRS-AKI patients. This improvement in renal function in terlipressin responders may also lead to a reduction in the model for the end-stage liver disease (MELD) score in patients awaiting liver transplantation. However, this may inadvertently cause terlipressin responders to face a disadvantage in the MELD score-based allocation system, resulting in a longer waiting time for liver transplantation [18]. Conversely, a recent study found that patients who did not respond to terlipressin treatment frequently developed severe AKI, necessitating renal replacement therapy. Moreover, a greater number of nonresponders progressed to chronic kidney disease after liver transplantation. On the other hand, terlipressin responders experienced longer waiting times for liver transplantation and had lower MELD scores at the time of transplantation. Importantly, terlipressin responders showed better transplant-free survival at day 30 and a lower incidence of chronic kidney disease or need for renal replacement therapy after liver transplantation. Thus, the response to terlipressin is a critical factor in determining both pre- and postliver transplantation outcomes [18,31].

The CONFIRM trial enrolled a cohort of 300 patients diagnosed with HRS-AKI [48]. This randomized controlled trial, conducted across multiple centers employing a doubleblind methodology, was carried out in North America. HRS-AKI was characterized as an abrupt deterioration in renal function, with serum creatinine (sCr) levels reaching or surpassing 2.25 mg/dL, and an actual or predicted twofold increase in sCr within a span of 2 weeks. This definition also stipulated the absence of any improvement in renal function (manifested by less than a 20% decrease in sCr within 48 h following both diuretic cessation and an albumin-fluid challenge). The trial encompassed adult patients afflicted by cirrhosis and ascites. Notably, both groups had a mean baseline MELD score of 33, and baseline sCr stood at a similar level of 3.5 mg/dL. The primary objective of the study centered on the verified reversal of HRS-AKI (VHRS-AKIR), denoted by two consecutive sCr measurements of ≤1.5 mg/dL, taken at intervals of at least 2 h, while the subjects sustained life without necessitating renal replacement therapy over the ensuing 10 days. The findings of the trial indicated a VHRS-AKIR rate of 29.1% in the terlipressin-treated group, contrasting with 15.8% in the placebo group (*p* = 0.01). However, a similar percentage of patients (approximately 28% in each group) remained alive at day 90 without necessitating a liver transplant. Terlipressin’s efficacy in reversing hepatorenal syndrome extended to patients with systemic inflammatory response syndrome (SIRS). For this subset, the trial reported a VHRS-AKIR rate of 33.43% in the terlipressin group compared to 6.3% in the placebo group. Furthermore, the demand for renal replacement therapy was substantially lower among participants receiving terlipressin [48].

In a multicenter chart review study encompassing 203 individuals afflicted by HRS-AKI, those who displayed a positive response to terlipressin exhibited enhanced survival rates at the three-month juncture. The median duration required for a favorable reaction to terlipressin was established at 8 days, with roughly 50% of patients achieving a comprehensive response [49]. In stark contrast, a mere 23% of the 22 patients subjected to alternative vasopressor agents achieved a comprehensive response. Adverse events were documented in around 25% of patients within the terlipressin-administered group and approximately 40% among those treated with other vasoconstrictors. However, a critical limitation of the study resided in the limited size of the cohort subjected to alternative vasoconstrictors. It is noteworthy that within this subset of 22 patients exposed to other vasoconstrictors, a substantial 60% had undergone vasopressin therapy. This circumstance complicates the establishment of definitive evidence concerning both effectiveness and safety.

As per a network meta-analysis encompassing 16 trials, a strong body of evidence emerged supporting the notion that the combination of terlipressin and albumin yields substantial improvements in short-term survival for patients grappling with type 1 hepatorenal syndrome. However, this improvement was not mirrored in individuals affected by type 2 hepatorenal syndrome [50]. Notably, the rates of complete reversal were elevated when terlipressin was coupled with noradrenaline alongside albumin compared to cases involving octreotide/midodrine or a placebo. The advantages were prominent in terms of short-term mortality, particularly when comparing terlipressin and albumin against the placebo. Nevertheless, a more recent network meta-analysis, encompassing 25 randomized clinical trials with a collective 1263 participants afflicted by cirrhosis and hepatorenal syndrome while evaluating 12 distinct interventions, has proposed the consideration of noradrenaline in conjunction with albumin as a potential alternative to terlipressin and albumin in forthcoming trials [51]. This analysis emphasized that noradrenaline displayed fewer adverse events. It is important to highlight that noradrenaline boasts a brief half-life of 1–2 min, requiring infusion through a central line and consistent monitoring in intensive care settings—attributes that can present significant drawbacks. Despite being a more cost-effective option and demonstrating comparable efficacy to terlipressin, noradrenaline did not offer any additional mortality benefits. However, for a thorough evaluation of the mortality benefits, further randomized clinical trials with adequately sized samples are imperative to compare noradrenaline against terlipressin [18].

## 8. Terlipressin: Tolerability and Pharmacovigilance

In the primary clinical trial, a notable 14% of patients who underwent terlipressin treatment encountered instances of severe or fatal respiratory failure in contrast to the 5% of patients receiving a placebo. Moreover, the utilization of terlipressin has been linked to a potential hazard of inducing cardiac, cerebrovascular, peripheral, or mesenteric ischemia. Hence, careful consideration is crucial, and the use of terlipressin should be refrained from in individuals with a history of severe cardiovascular ailments, cerebrovascular disease, or ischemic conditions. Additionally, it is important to recognize that administering terlipressin to pregnant women could potentially lead to harm to the fetus due to its mode of action. This is attributed to the fact that the drug triggers uterine contractions and endometrial ischemia in both human and animal subjects. As a result, if terlipressin is administered during pregnancy, patients need to be informed about the potential risks posed to the developing fetus. Furthermore, it is noteworthy that terlipressin-associated adverse reactions could render a patient unsuitable for liver transplantation, especially if they are already listed as candidates. For individuals with a high priority for liver transplantation (MELD ≥ 35), the potential risks linked with terlipressin might outweigh the perceived benefits. In light of these considerations, it is important to emphasize that terlipressin is contraindicated in patients who are experiencing hypoxia or a worsening of respiratory symptoms. Moreover, its use should be avoided in patients grappling with ongoing coronary issues [25,28,48].

The safety profile of terlipressin was evaluated within the CONFIRM trial. The mean daily dose administered averaged at 3.1 mg, while the typical duration of treatment spanned 6.2 days. Within the cohort receiving terlipressin, 12.0% (24 out of 200) discontinued treatment due to adverse events, in contrast with 5.1% (5 out of 99) of patients who were on a placebo and stopped treatment for the same reasons. Notably, the most frequently observed adverse reactions leading to the discontinuation of terlipressin were respiratory failure, abdominal pain, and instances of intestinal ischemia/obstruction. Table 2 offers an overview of the adverse reactions occurring more frequently among patients receiving terlipressin when compared to those administered a placebo. These reactions were apparent in a minimum of 4% of patients subjected to terlipressin treatment during the CONFIRM trial. The most commonly witnessed adverse reactions in patients treated with terlipressin, affecting 10% or more of the patients, encompassed abdominal pain, nausea, respiratory failure, diarrhea, and dyspnea. Additionally, adverse reactions garnered from postmarketing experiences with terlipressin across the globe involve hyponatremia, headache, skin necrosis, and gangrene. Given that these reactions are reported on a voluntary basis and stem from a variable population size, ascertaining their frequency or establishing a direct causal connection to terlipressin exposure is not consistently feasible [25,28,48].

In relation to the utilization of terlipressin among particular groups, currently, there is an absence of available data concerning the presence of terlipressin in human or animal milk, its potential effects on infants being breastfed, or its influence on milk production. The safety and efficacy of terlipressin have not been established for pediatric patients. Among clinical trials, out of the overall pool of patients treated with terlipressin, a subgroup of 55 individuals (16%) were aged 65 years or above. No significant contrasts in safety or efficacy emerged between these older participants and their younger counterparts. While previous clinical experiences have not unveiled any noteworthy variances in responses between older and younger patients, it is important to consider that certain elderly individuals might exhibit heightened sensitivity to the medication. Nonetheless, specific dose adjustments are not deemed necessary for patients with impaired liver function. Examinations on the carcinogenic potential of terlipressin have not been conducted, precluding an assessment of its propensity to induce cancer. Through assessments like the in vitro bacterial reverse mutation assay, the in vivo mouse micronucleus assay, and the in vitro mammalian cell (CHO) chromosome aberration assay, terlipressin was ascertained to be devoid of mutagenic and clastogenic properties. Furthermore, no animal studies have been executed to evaluate terlipressin’s impact on fertility [25].

## 9. Conclusions

Terlipressin, a vasopressin receptor agonist, obtained approval from the US FDA in 2022 for the enhancement of kidney function in adult patients dealing with hepatorenal syndrome marked by swift declines in kidney function. Its usage is limited for patients whose serum creatinine levels exceed 5 mg/dL [52]. Drawing upon the findings of the CONFIRM trial, the combination of terlipressin and albumin demonstrated greater efficacy compared to the pairing of a placebo and albumin. This was particularly evident in achieving the verified reversal of hepatorenal syndrome in individuals afflicted by decompensated cirrhosis and HRS-AKI. Notably, the use of terlipressin was associated with serious adverse events, including instances of respiratory failure [28,48].

Historically, hepatorenal syndrome has been characterized by a rapid deterioration in renal function and a correspondingly elevated mortality rate. Managing this acute condition presents considerable challenges. Various medications, such as octreotide, midodrine, and dopamine, in conjunction with albumin, have been employed in attempts to manage hepatorenal syndrome. Yet, none have demonstrated the capability to halt the progression of declining renal function. With terlipressin’s mode of action in mind, the focus of management has shifted toward impeding the initial phases of vasodilation, which ultimately contributes to the rapid decline in renal function. This shift was substantiated by the CONFIRM trial and other studies involving patients with HRS-AKI [28,41,48,53]. However, there are critical considerations to bear in mind. Vigilant monitoring of respiratory status in patients is imperative. The management of intravascular volume overload should be carefully executed, and therapy adjustments should be made judiciously. It is also noteworthy that adverse reactions linked to terlipressin might render patients ineligible for liver transplantation. Given that terlipressin functions as a vasoconstrictor, the potential for inducing ischemic events—cardiac, peripheral, or mesenteric—exists, necessitating the possibility of dose interruption or discontinuation [54,55].

## Figures and Tables

**Table 1.** Clinical trials of terlipressin in patients diagnosed with cirrhosis and hepatorenal syndrome.

Study No. of Participants	Dose of Terlipressin	Comparator	Reversal and Survival Benefit with Terlipressin	Ref.
**HRS-AKI**				
Moreau R et al. (2002), *N* = 99	3.2 ± 1.3 mg/day infusion	None	64% reversal	[32]
Solanki P et al. (2003), *N* = 12 in each group	1 mg every 12 h	Placebo	41.67% reversal and survival benefit on day 15	[33]
Neri S et al. (2008), *N* = 26 in each group	1 mg every 8 h for 5 days, then 0.5 mg every 8 h for 2 weeks with albumin	Albumin	80% reversal (vs. 19%)	[34]
Sharma P et al. (2008), *N* = 20 in each group	0.5 mg every 6 h. increasing every third day (in case of no response) to 2 mg every 6 h	Noradrenaline/albumin	50% reversal in each group Survival was similar on day 15	[35]
Nazar A et al. (2010), *N* = 39	0.5 to 1 mg every 4 h for 3 days. The dose increased to 2 mg every 4 h with albumin if sCr had not decreased >25%	None	46% reversal	[36]
Singh V et al. (2012), *N* = 23 in each group	0.5 mg every 6 h. increasing every third day (in case of no response) to 2 mg every 6 h	Noradrenaline/albumin	39% reversal (vs. 43.4%) Survival was similar in both groups	[37]
Ghosh S et al. (2013), *N* = 23 in each group	0.5 mg every 6 h, increasing every third day when no response achieved to 2 mg every 6 h with albumin	Noradrenaline/albumin	74% reversal (vs. 74%) No survival benefit	[38]
Cavallin M et al. (2015), *N* = 27 in terlipressin and 22 in the other group	3 mg infusion for 24 h, increased to 12 mg over 24 h if no response achieved with albumin	Midodrine/octreotide/albumin	70.4% reversal (vs. 28.6%) No survival benefits	[39]
Goyal O et al. (2016), *N* = 41	0.5–2 mg every 6 h with albumin	Noradrenaline/albumin	45% reversal (vs. 47.6%)	[40]
Sanyal A et al. (2017), *N* = 308 patients (terlipressin = 153; placebo = 155)	1 mg every 6 h. Dose doubled to 2 mg every 6 h when no significant improvement achieved	Placebo	27% reversal (vs. 14%) Survival benefit on day 90	[41]
Saif RU et al. (2018), *N* = 30 in each group	0.5 mg every 6 h with maximum dose of 4 mg every 6 h with albumin	Noradrenaline/albumin	57% reversal (vs. 53%) No survival benefit	[42]
Wong F et al. (2021), *N* = 300 (199 were assigned to the terlipressin + albumin group and 101 to the placebo + albumin group)	1 mg of terlipressin was administered IV every 5.5 to 6.5 h. Patient could receive up to 2 mg every 6 h on day 4 if no sufficient response achieved	Placebo/albumin	(1) 32% verified reversal (vs. 17%) (2) Reversal without renal replacement therapy by day 30 was 34% (vs. 17%) (3) Reversal among patients with systemic inflammatory response syndrome was 37% (vs. 6%) (4) 26% verified reversal (vs. 17%) without recurrence by day 30 (5) At day 90, liver transplantations had been performed in 23% (vs. 29%) and death occurred in 51% (vs. 45%)	[28]
**HRS-AKI and HRS-NAKI**				
Uriz J et al. (2000), *N* = 9	0.5 mg every 4 h. Increased every third day to 1 mg every 4 h and 2 mg every 4 h if no adequate response	None	78% reversal	[43]
Ortega R et al. (2002), *N* = 21 patients (five were HRS-NAKI)	0.5–2 mg every 4 h with albumin	None	57% reversal (vs. 10%)	[44]
Alessandria C et al. (2007), *N* = 22 (12 in terlipressin group)	1–2 mg every four hours	Noradrenaline/albumin	83% reversal (vs. 70%) No survival benefit	[45]
Martin-Llahi M et al. (2008), *N* = 23 in each group	1 mg every 4 h for 3 days. If no adequate response, the dose was increased to 2 mg every 4 h	Albumin	43.5% reversal (vs. 8.7%) No survival benefit at day 90	[46]
Nguyen-Tat M et al. (2019), *N* = 106	4 mg/day IV infusion	None	46–48% reversal	[47]

**Table 2.** Adverse reactions reported by 4% or more of patients treated with terlipressin.

Adverse Event	Placebo %	Terlipressin %
Abdominal pain	6.1	19.5
Nausea	10.1	16.0
Respiratory failure	7.1	15.5
Diarrhea	7.1	13.0
Dyspnea	5.1	12.5
Fluid overload	3.0	8.5
Pleural effusion	0	5.5
Sepsis	1.0	5.5
Bradycardia	0	5.0
Ischemia-related events (skin discoloration, cyanosis, ischemia, and intestinal ischemia)	0	4.5

Reprint from Refs. [25,30].
